# Targeted lipidomics analysis identified altered serum lipid profiles in patients with polymyositis and dermatomyositis

**DOI:** 10.1186/s13075-018-1579-y

**Published:** 2018-05-02

**Authors:** Joan Raouf, Helena Idborg, Petter Englund, Helene Alexanderson, Maryam Dastmalchi, Per-Johan Jakobsson, Ingrid E. Lundberg, Marina Korotkova

**Affiliations:** 1 Division of Rheumatology, Department of Medicine, Karolinska Institutet, Karolinska University Hospital, Center for Molecular Medicine, Stockholm, Sweden; 2 Department of Environmental Science and Analytical Chemistry, Stockholm University, Stockholm, Sweden

**Keywords:** Lipidomics, Fatty acids, Phospholipids, Polymyositis, Dermatomyositis, Immunosuppressive treatment

## Abstract

**Background:**

Polymyositis (PM) and dermatomyositis (DM) are severe chronic autoimmune diseases, characterized by muscle fatigue and low muscle endurance. Conventional treatment includes high doses of glucocorticoids and immunosuppressive drugs; however, few patients recover full muscle function. One explanation of the persistent muscle weakness could be altered lipid metabolism in PM/DM muscle tissue as we previously reported. Using a targeted lipidomic approach we aimed to characterize serum lipid profiles in patients with PM/DM compared to healthy individuals (HI) in a cross-sectional study. Also, in the longitudinal study we compared serum lipid profiles in patients newly diagnosed with PM/DM before and after immunosuppressive treatment.

**Methods:**

Lipidomic profiles were analyzed in serum samples from 13 patients with PM/DM, 12 HI and 8 patients newly diagnosed with PM/DM before and after conventional immunosuppressive treatment using liquid chromatography tandem mass spectrometry (LC-MS/MS) and a gas-chromatography flame ionization detector (GC-FID). Functional Index (FI), as a test of muscle performance and serum levels of creatine kinase (s-CK) as a proxy for disease activity were analyzed.

**Results:**

The fatty acid (FA) composition of total serum lipids was altered in patients with PM/DM compared to HI; the levels of palmitic (16:0) acid were significantly higher while the levels of arachidonic (20:4, n-6) acid were significantly lower in patients with PM/DM. The profiles of serum phosphatidylcholine and triacylglycerol species were changed in patients with PM/DM compared to HI, suggesting disproportionate levels of saturated and polyunsaturated FAs that might have negative effects on muscle performance. After immunosuppressive treatment the total serum lipid levels of eicosadienoic (20:2, n-6) and eicosapentaenoic (20:5, n-3) acids were increased and serum phospholipid profiles were altered in patients with PM/DM. The correlation between FI or s-CK and levels of several lipid species indicate the important role of lipid changes in muscle performance and inflammation.

**Conclusions:**

Serum lipids profiles are significantly altered in patients with PM/DM compared to HI. Moreover, immunosuppressive treatment in patients newly diagnosed with PM/DM significantly affected serum lipid profiles. These findings provide new evidence of the dysregulated lipid metabolism in patients with PM/DM that could possibly contribute to low muscle performance.

**Electronic supplementary material:**

The online version of this article (10.1186/s13075-018-1579-y) contains supplementary material, which is available to authorized users.

## Background

Polymyositis (PM) and dermatomyositis (DM) are chronic autoimmune disorders characterized by skeletal muscle weakness, fatigue and damage. The major histopathological features of these diseases include infiltration of inflammatory cells in skeletal muscle tissue and muscle fiber degeneration and regeneration [1, 2]. Furthermore, immune mechanisms including immune cell infiltration, major histocompatibility complex (MHC) class I molecule expression in muscle cells, production of pro-inflammatory mediators and presence of autoantibodies are implicated in PM/DM pathogenesis and might contribute to muscle weakness [3–6].

Conventional treatment of PM and DM is based on immunosuppression including high doses of glucocorticoids (GC) often in combination with other immunosuppressive drugs [1, 2], but despite clinical improvement, only a few patients fully restore previous muscle strength. There is accumulating evidence that non-immune mechanisms such as endoplasmic reticulum (ER) stress and capillary-loss-induced hypoxia are important contributors to muscle weakness in patients with inflammatory myopathies [7, 8]. ER stress-induced aberrant reactive oxygen species (ROS) generation, mitochondrial dysfunction and myokine induction are implicated in skeletal muscle dysfunction [8]; moreover, there is cross-talk between ER stress and dysregulated lipid metabolism [9]. Changes in muscle lipid metabolism may be caused by ER stress, chronic inflammation or immunosuppressive treatment as suggested by recent reports [9, 10] and in turn lead to altered lipid profiles contributing to persistent muscle impairment. There is growing understanding of an important role of various lipids such as fatty acids (FA), phospholipids, sphingolipids and prostaglandins in the regulation of skeletal muscle growth and functions [11–14]. For instance, deviations in the phospholipid levels or FA composition are associated with impaired muscle performance while dietary supplementation with unsaturated FA improves skeletal muscle mass and strength [14–18].

Recent lipidomic studies have demonstrated that serum/plasma phospholipids, lysophospholipids, neutral lipids and FA are altered in chronic inflammatory diseases including rheumatoid arthritis (RA), psoriasis, type 2 diabetes mellitus and obesity [19–22]. The changes in lipid metabolism in these diseases might be induced by inflammatory responses via various mechanisms [23]. Information on lipid profiles in patients with PM or DM is very limited. A few reports suggest altered serum levels of low-density lipoprotein (LDL)-cholesterol, high-density lipoprotein (HDL)-cholesterol, total cholesterol and triglycerides indicating changes in lipid metabolism in these diseases [24–26]. The dyslipidemia was associated with disease activity in one study and may reflect an interaction between inflammation and lipid metabolism [26]. These results suggest that detailed characterization of lipid profiles in patients with PM or DM may add to our understanding of molecular mechanisms leading to persisting low muscle performance.

To identify lipid changes that may play a role in pathogenesis of persisting muscle weakness in patients with PM and DM, we examined lipid profiles in serum from patients with PM and DM using a targeted lipidomic approach. Lipid profiles were investigated in patients with PM and DM and compared to healthy individuals (HI) and in response to conventional immunosuppressive treatment.

## Methods

### Patients and serum samples

This study comprises two parts, one cross-sectional (13 patients with PM and DM) and one longitudinal (8 patients with PM and DM). The patients with PM or DM fulfilled the Bohan and Peter classification criteria for possible, probable or definite disease [27, 28]. Patients with inclusion body myositis and immune necrotizing myopathy (IMNM) were excluded based on clinical manifestations and muscle biopsy features. Myositis-specific autoantibodies (MSA) were tested by immunoprecipitation in collaboration with Dr. Mimori, Kyoto, Japan [29] and for anti-3-hydroxy-3-methylglutaryl-coenzyme A reductase (anti-HMGCR) antibodies by Dr. Mammen, Baltimore, USA [30]. The remaining serum was tested in the Clinical Immunology Laboratory, Karolinska University Hospital, as routine analyses for antinuclear antibodies (ANA), anti-Sjogren’s syndrome-related antigen A (anti-SSA), anti-Ro52, anti-Sjogren’s syndrome-related antigen B (anti-SSB), anti-U1RNP, anti-double stranded DNA (anti-dsDNA) and anti-Jo1 antibodies. All 21 patients were tested with at least one of these methods. There were 18 patients who were seropositive for at least one autoantibody: anti-Jo-1 (2 with PM, 1 with DM), anti-TIF1γ (1 with DM, 1 with PM), anti-Mi-2 (1 with DM), anti-MDA5 (1 with DM), anti-HMGCR (1 with DM), anti-SRP (1 with PM), anti-SSA (1 with PM, 1 with DM), anti-Ro52 (2 with DM), anti-RNP (2 with PM) or anti-nuclear antibodies (4 with DM). Three patients with diagnosis of PM were negative in the antibody tests; in these patients the diagnosis of PM was supported by muscle biopsy findings of endomysial inflammatory infiltrates surrounding non-necrotic fibers and in two patients there were also perivascular infiltrates, but none had signs of any other myopathy. None of the patients had histopathological features compatible with necrotizing myopathy.

Clinical data on comorbidities that may affect metabolism and lipid profiles were retrieved by retrospective review of patients’ records. None of the 21 patients had diabetes mellitus. One patient had received thyroid hormone replacement for several years and normal serum levels of thyroid stimulating hormone (TSH) at the time of blood sampling, and thyroid disease was excluded by normal TSH in 19/20 patients (one TSH value was missing).

Lipid profiles were compared between patients with PM or DM and HI in the cross-sectional study. Stored serum from 13 consecutive patients with PM or DM (median age 46 years, range 25–60 years; 7 women and 6 men; disease duration median 12 months, range 1–144 months) were analyzed. At the time of serum sampling eight patients were on treatment with prednisolone with a median daily dose of 15 mg/day. Six patients had an additional immunosuppressive drug (ciclosporin, chloroquine, azathioprine or cyclophosphamide); one patient was treated with azathioprine only and four patients were untreated.

Serum samples from 12 HI (6 women, 6 men, median age 45, range 25–60 years) matched by age and gender were obtained from the biobank at the Division of Rheumatology, Karolinska Institutet and included as controls.

The longitudinal study included eight consecutive patients, newly diagnosed with PM or DM, untreated (*n* = 5) or treated for < 2 weeks (n = 3) (median age 61.5 years, range 49–80 years; 7 women, 1 man) from whom two serum samples were collected, one before or within 2 weeks of prednisolone treatment and a second serum sample approximately 8 months after starting immunosuppressive treatment. Treatment consisted of oral prednisolone (60 mg/day) with slowly tapering doses to minimum of 7.5 mg/day and all patients received an additional immunosuppressive drug (methotrexate, azathioprine or cyclophosphamide) as decided by the treating physician.

Whole blood was obtained from non-fasting individuals by venipuncture into 5-ml vacutainer serum separator tubes with clot activator (Becton Dickinson). According to the manufacturer’s instructions the samples were centrifuged, and serum was separated, immediately frozen and stored at − 80 °C until analysis.

### Clinical and laboratory assessment

Laboratory and clinical outcome measurements were retrieved from medical records. As a test of muscle performance, a modified Functional Index (FI) was applied including the hip flexion and the step test with a maximum score of 100% [31]. An increase ≥ 15% was defined as an improvement. As a proxy for disease activity, serum levels of total creatine phosphokinase (s-CK) were measured using an enzymatic rate analysis and spectrophotometric method (the Beckman DxC system) at the Department of Clinical Chemistry, Karolinska University Hospital, Sweden. Reference values were 0.6–3.5 μkat/L for women and 0.8–6.7 μkat/L for men.

### Sample extraction for lipid profiling

All samples were kept on ice during the extraction of serum lipids and FA. In short, 0.5 ml frozen serum was thawed at 4 °C and centrifuged at 1000 × g for one minute. 100 μl serum was transferred to Eppendorf tubes and 10 μl of internal standard (10 ng/μl of triheptadecanoin in chloroform (CHCl_3_)) was added to each sample. Furthermore, a 750-μl mixture of methanol (MeOH) and CHCl_3_ (2:1 v:v) was added to each sample, vortexed for 10 s and sonicated for 10 min. Samples were spun down for 5 min at 2000 × g. The organic phase, containing lipids, was removed and saved in Eppendorf tubes. The sample was then re-extracted by the same procedures as described above. The second extract was combined with the first extract for each sample and evaporated in a SpeedVac (ScanVac, Labogene, Denmark). The lipids obtained were dissolved and vortexed in 1 ml MeOH/CHCl_3_ (2:1 v:v) and further divided into two portions each containing 500 μl and stored at − 80 °C until analysis of FA of total lipids and targeted lipidomics analysis, respectively.

### Analysis of FA composition of total serum lipids

Extracted lipids were transformed into FA methyl esters (FAMEs) according to Idborg et al. [32]. Briefly, 2 ml hexane, 1 ml MeOH and 1 ml boron trifluoride (BF_3_) solution was added to the first portion of the dissolved lipids. Samples were then gradually purged with nitrogen, tightly sealed and incubated at 95 °C for 1 h. After incubation, an equivalent amount of Milli-Q water was added, and samples were centrifuged at 500 rpm for 10 min, the top phase was collected, and the bottom phase was re-extracted with hexane. The samples were analyzed with a gas-chromatography flame ionization detector (GC-FID) (YL6100, Younglin Lin Instrument Co., Ltd., Korea), together with an HT300A autosampler (Younglin Lin Instrument Co., Ltd., Korea), equipped with a 30 m × 0.32 mm i.d. × 0.25 μm film thickness, J.W. Scientific DB-Wax column (Agilent Technologies Inc., Santa Clara, CA, USA) using the analytical conditions described in Idborg et al. [32]. A calibration curve was plotted in the range from 2.5 to 100 ng/μl, and the individual relative response factors were calculated by comparing the areas of the analytes to the ones of the internal standard. Two replicate injections of each sample were performed, and the results were expressed in weight percentage, as a mean value ± standard deviation (SD) of the total identified FAMEs. The mean values were used to calculate fold change.

### Targeted lipidomics analysis

Analysis of several lipid classes, e.g., triacylglycerols (TG), phospholipids, sphingolipids, and lysophospholipids was performed using liquid chromatography tandem mass spectrometry (LC-MS/MS). In brief, the second portion of the dissolved lipids was analyzed by Waters 2795 HPLC (Waters Corporation, MA, USA) coupled to a triple quadrupole mass spectrometer (Acquity TQ Detector, Water Corporation). Samples were injected into a Hypersil GOLD Phenyl column (150 mm × 2.1 mm i.d, 3 μm particle size, ThermoFisher Scientific, USA) with a 45-min stepwise linear gradient: 5 mM ammonium acetate in MilliQ water was used as mobile phase A and 5 mM ammonium acetate in methanol was used as mobile phase B. The gradient started at 20% B for 2 min followed by an increase from 80% to 99% B in 18 min and then stayed at 99% for 15 min. The re-equilibration step was performed going back to 80% in 1 min and equilibrate for 9 min. One 20-μl aliquot was injected for detection of phosphatidylcholine (PC) and sphingomyelin (SPH) species by multiple reaction monitoring (MRM) mode, and one 20 μl aliquot was injected detecting phosphatidylethanolamine (PE) and lysophosphatidylcholine (LPC) species by MRM. Integration of peaks was performed by MassLynx software, version 4.1. The relative abundance of lipid species was calculated using area percentage.

### Statistical analysis

Univariate statistical analysis was performed using the Mann-Whitney U test and Wilcoxon’s signed rank test. The Spearman rank correlation test was used to test correlation between the clinical parameters and the serum levels of specific lipid metabolites. The strength of the Spearman correlation is referred to as weak (Rho <0.39), moderate (Rho = 0.4–0.59) or strong (Rho >0.60) [33]. Differences with *p* values less than 0.05 were considered statistically significant.

Principal component analysis (PCA) and orthogonal projection to latent structures (OPLS) were performed utilizing SIMCA P+ version 12 (MKS Data Analytics Solution, Umeå, Sweden). Prior to PCA/OPLS the data were mean-centered and scaled to unit variance.

## Results

### FA composition of serum total lipids in patients with PM/DM and HI

First, as a screening test we examined the FA composition of total serum lipids in patients with PM/DM and HI using GC-FID. A total of 24 FA were measured, and 11 FA were detected in total serum lipids in both groups (Table 1). The FA profiles were altered in patients with PM/DM compared to HI (Table 1). Significant differences were observed in the levels of the palmitic (16:0) acid (PA), which were higher (*p* < 0.05) in patients with PM/DM in comparison with HI. In contrast, the levels of the arachidonic (20:4, n-6) acid (AA) were significantly lower (*p* < 0.05) in patients with PM/DM. In addition, the total levels of saturated fatty acids (SFA) were higher (*p* = 0.091) while the levels of docosahexaenoic (22:6, n-3) acid were lower (*p* = 0.073) in patients with PM/DM compared to HI. No significant differences were detected in the total levels of monounsaturated FA (MUFA), polyunsaturated FA (PUFA), n-3 PUFA or n-6 PUFA in patients with PM/DM compared to matched HI (Table 1).

**Table 1:** Fatty acid composition of total serum lipids from patients with polymyositis and dermatomyositis compared to healthy individuals

FA	Healthy individuals,weight %	Patients,weight %
C14:0	1.6 ± 0.8	2.1 ± 0.7
C16:0	21.6 ± 5.2	24.5 ± 3.0*
C16:1	1.8 ± 0.7	2.1 ± 0.8
C18:0	9.8 ± 1.0	8.9 ± 1.3
C18:1	24.7 ± 3.0	26.5 ± 4.6
C18:2(n-6)	27.9 ± 4.4	25.6 ± 5.1
C18:3(n-3)	1.4 ± 0.7	1.2 ± 0.5
C20:3(n-6)	1.3 ± 0.4	1.1 ± 0.5
C20:4(n-6)	5.4 ± 1.1	4.3 ± 1.3*
C20:5(n-3)	2.2 ± 1.2	1.8 ± 1.4
C22:6(n-3)	2.4 ± 0.8	1.9 ± 0.7
SFA	32.9 ± 4.9	35.5 ± 3.0
MUFA	26.5 ± 3.4	28.7 ± 5.1
PUFA	40.5 ± 5.8	35.8 ± 7.3
n-3 PUFA	5.9 ± 1.7	4.9 ± 2.0
n-6 PUFA	34.6 ± 4.9	30.9 ± 6.3

Values given as mean ± SD

*FA* fatty acids, *SFA* saturated fatty acids, *MUFA* monounsaturated fatty acids, *PUFA* polyunsaturated fatty acids

*p < 0.05 for patients versus healthy individuals

### Targeted lipidomics analysis of serum lipids from patients with PM/DM and HI

**Fig. 1:**
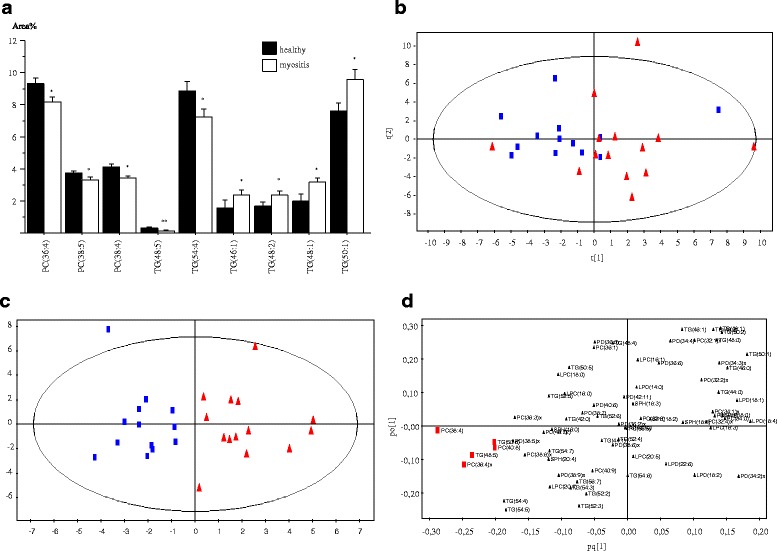
**a** Serum lipid species in patients with polymyositis or dermatomyositis compared to healthy individuals. PC, phosphatidylcholine; TG, triacylglycerol. Results are expressed as percentage area (area %) (mean ± SE): *p < 0.05, **p < 0.01, for patients with polymyositis (PM)/dermatomyositis (DM) versus healthy individuals. **b**, **c** Score plots from unsupervised clustering by principal component analysis (PCA) (**b**) and supervised clustering by orthogonal projection to latent structures (OPLS) (**c**) are shown. Healthy individuals are represented by blue squares and patients with PM/DM by red triangles. **d** The corresponding loading plot obtained from OPLS analysis. Lipids important for the separation of patients with PM/DM from controls are highlighted in red

To gain in-depth insight into lipid metabolism in patients with PM/DM compared to HI, we applied LC-MS/MS-based targeted lipidomics to analyze serum PC, LPC, TG and SPH levels in patients and HI. In total, 72 lipid species were identified including 30 PC, 11 LPC, 25 TG and 6 SPH species (Additional file 1: Table S1). Out of 72 lipid species, 9 were significantly altered and within the PC class, the levels of species containing PUFA such as PC(36:4), PC(38:5) and PC(38:4) were lower in patients with PM/DM than in HI (Fig. 1a, Additional file 1: Table S1). Among the TGs, the species containing SFA and MUFA, such as TG(46:1), TG(48:2), TG(48:1) and TG(50:1), were higher in patients with PM/DM compared to HI while species containing PUFA, such as TG(48:5) and TG(54:4) were lower (Fig. 1a, Additional file 1: Table S1). Both within PC and TG, the ratio of lipid species containing SFA and MUFA to those containing PUFA were significantly higher in patients with PM/DM compared to HI. There were no significant differences in the LPC and SPH levels between patients with PM/DM and HI (Additional file 1: Table S1).

Multivariate analysis (MVA) using PCA showed a modest separation of patients with PM/DM and HI (Fig. 1b). Supervised clustering was performed using OPLS to distinguish which variables most prominently separate HI from patients with PM/DM. Fig. 1 shows the score plot (Fig. 1c) and the corresponding loading plot (Fig. 1d). Variables important for the separation between HI and patients with PM/DM were selected based on a combination of variable influence in projection (VIP >1.5) and scaled loadings (*p*(corr) >|0.5|) in the OPLS model. The important variables were PC(38:4), PC(36:4), PC(40:8), TG(48:5) and TG(50:6) (highlighted in the loading plot in red, Fig. 1d).

### Effects of immunosuppressive treatment on FA composition of total serum lipids from patients with PM/DM

Next we analyzed the FA composition of total serum lipids in patients with PM/DM before and after immunosuppressive treatment. The levels of the eicosadienoic (20:2, n-6) acid (EDA) and the eicosapentaenoic (20:5, n-3) acid (EPA) were significantly increased (*p* < 0.05) after treatment (Table 2). No significant changes were observed in total levels of SFA, MUFA, PUFA, n-3 FA or n-6 FA in patients after treatment (Table 2).

**Table 2:** Fatty acid composition of total serum lipids from patients with polymyositis and dermatomyositis before and after immunosuppressive treatment

FA	Before treatment, weight %	After treatment,weight %
C14:0	0.96 ± 0.48	1.33 ± 0.33
C16:0	22.78 ± 1.98	23.18 ± 1.60
C16:1	1.85 ± 0.84	2.06 ± 0.93
C18:0	10.76 ± 1.44	9.96 ± 1.33
C18:1	25.34 ± 3.53	24.02 ± 2.92
C18:2(n-6)	21.98 ± 3.34	23.02 ± 3.11
C18:3(n-6)	0.04 ± 0.12	0.17 ± 0.20
C18:3(n-3)	1.81 ± 1.03	2.37 ± 0.65
C20:1	0.3 ± 0.32	0.13 ± 0.26
C20:2(n-6)	0.1 ± 0.13	0.46 ± 0.37*
C20:3(n-6)	1.27 ± 0.36	1.31 ± 0.34
C20:4(n-6)	5.83 ± 1.61	4.96 ± 1.07
C20:5(n-3)	1.25 ± 0.44	1.91 ± 0.65*
C22:0	0.2 ± 0.45	0.2 ± 0.17
C22:5(n-3)	1.0 ± 0.44	1.18 ± 0.18
C22:6(n-3)	4.38 ± 1.77	3.55 ± 0.70
SFA	34.7 ± 2.6	34.9 ± 2.7
MUFA	26.4 ± 3.4	27.5 ± 4.5
PUFA	38.9 + 3.4	37.6 + 4.9
n-3 PUFA	9.0 ± 1.6	8.4 ± 1.9
n-6 PUFA	29.9 ± 2.8	29.2 ± 4.1

Values given as mean ± SD

*FA* fatty acids, *SFA* saturated fatty acids, *MUFA* monounsaturated fatty acids, *PUFA* polyunsaturated fatty acids

*p < 0.05 for after treatment versus before treatment

### Targeted lipidomics analysis of serum lipids from patients with PM/DM before and after immunosuppressive treatment

**Fig. 2:**
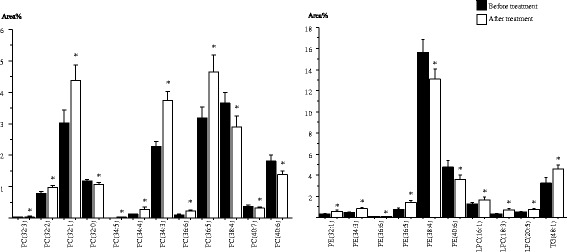
Serum lipid species in patients with polymyositis or dermatomyositis before and after immunosuppressive treatment. PC, phosphatidylcholine; PE, phosphatidylethanolamine; LPC, lysophosphatidylcholine; TG, triacylglycerol. Results are expressed as percentage area (Area %) (mean ± SE): *p < 0.05 for after treatment versus before treatment

Using a more comprehensive LC-MS/MS-based targeted lipidomics approach, we identified a total of 97 serum lipid species including 30 PCs, 29 PE, 10 LPCs and 24 TGs in patients with PM/DM before and after immunosuppressive treatment (Additional file 1: Table S2). Twenty-two lipid species were altered in response to immunosuppressive treatment. After treatment, significant differences (*p* < 0.05) were observed in the levels of 12 PC species. The levels of eight PCs, i.e., PC(32:3), PC(32:2), PC(32:1), PC(34:5), PC(34:4), PC(34:3), PC(36:6) and PC(36:5), were increased in patients with PM/DM in response to immunosuppressive treatment (Fig. 2, Additional file 1: Table S2). The levels of four species, PC(32:0), PC(38:4), PC(40:7) and PC(40:6), were reduced after treatment. Within the PE class, the levels of PE(32:1), PE(34:3), PE(36:6) and PE(36:5) were also significantly higher (*p* < 0.05) while PE(38:4) and PE(40:6) levels were lower after treatment (Fig. 2, Additional file 1: Table S2). After treatment the serum levels of LPC(16:1), LPC(18:3), LPC(20:5) and TG(48:1) were significantly increased (Fig. 2, Additional file 1: Table S2).

**Fig. 3:**
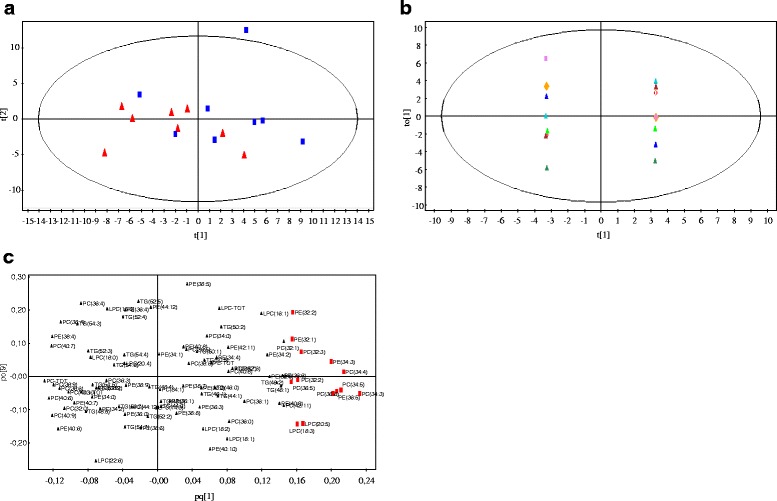
Results of multivariate analysis of the lipid species data obtained from patients with polymyositis (PM)/dermatomyositis (DM) before and after immunosuppressive treatment. **a** Score plot from unsupervised clustering by principal component analysis (PCA). Samples from patients with PM/DM before treatment are shown as blue squares and after treatment as red triangles. **b** Score plot visualizing separation of the samples from patients before and after treatment based on supervised clustering (OPLS). Paired before-treatment and after-treatment samples are shown in the same color. **c** The corresponding loading plot obtained from OPLS. Lipids important for the separation of patients before and after treatment are highlighted in red

**Fig. 4:**
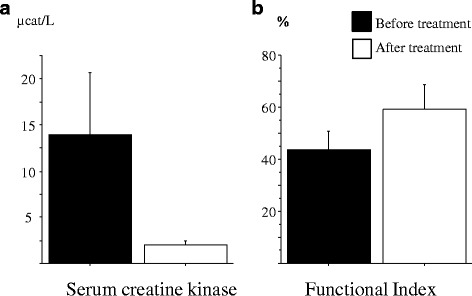
**a** Serum creatine kinase levels and **b** functional index (FI) in patients with polymyositis or dermatomyositis before and after immunosuppressive treatment. Results are expressed as μcat/L and percentage, respectively (mean ± SE)

The results of MVA did not separate patients before and after treatment in a PCA score plot of the first two dimensions (Fig. 3a). Using OPLS we performed supervised clustering of lipidomic profiles obtained from patients with PM/DM before and after treatment, and Fig. 3. shows the score plot (Fig. 3b) and the corresponding loading plot (Fig. 3c). Analysis of lipid species selected based on a combination of VIP and scaled loadings (*p*(corr)) in the OPLS model demonstrated that the most important variables for the separation between before and after treatment were PC(36:6), PC(36:5), PC(34:5), PC(34:4), PC(34:3), PC(32:3), PC(32:2), PE(36:5), PE(34:3), PE(32:2), PE(32:1), LPC(20:5) and LPC(18:3) (Fig. 4c, highlighted in the loading plot in red).

### Correlation between lipid markers and clinical parameters

As a proxy for disease activity, s-CK levels trended toward reduction after immunosuppressive treatment (median 7.6, range 0.3–50 μkat/L vs median 1.8, range 0.3–4.2 μkat/L, *p* = 0.115, before and after treatment, respectively) (Fig. 4a). FI was improved in three out of eight patients after treatment, but at the group level no significant change was seen (median 43%, range 16–67% vs median 60.8%, range 11–84%, before and after treatment, respectively) (Fig. 4b).

A Spearman’s correlation test was performed to assess the relationship between serum lipid markers and clinical parameters in patients with PM/DM before and after immunosuppressive treatment. Before treatment positive correlation was observed between the levels of s-CK and the levels of PE(36:1) (Rho = 0.848; *p* = 0.037) and between FI and the levels of TG(52:5) (Rho = 0.810; *p* = 0.032). After treatment, positive correlation was detected between s-CK and the levels of PE(38:7) (Rho = 0.813; *p* = 0.046) and TG(56:7) (Rho = 0.857; *p* = 0.035). Negative correlation was observed between s-CK and the levels of LPC(20:4) (Rho = − 0.964; *p* = 0.018). There was negative correlation between FI and the levels of PC(34:2) (Rho = − 0, 857; *p* = 0.035); positive correlation between FI and the levels of PC(36:4) (Rho = 0.964; *p* = 0.018), PC(38:4) (Rho = 0.821; *p* = 0.044), TG(50:6) (Rho = 0.830; *p* = 0.042) and TG(52:5) (Rho = 0.857; *p* = 0.035) after treatment.

## Discussion

In the present study we have provided a detailed characterization of the serum lipid profiles in patients with PM/DM for the first time. Using a targeted lipidomics approach we have demonstrated that serum lipid profiles are altered in patients with PM/DM compared with HI. Moreover, immunosuppressive treatment of patients newly diagnosed with PM/DM significantly affected serum lipid profiles. We detected significantly higher serum levels of PA in patients with PM/DM compared to HI. PA is the most abundant systemic SFA and has been linked to inflammation [34, 35], insulin resistance [36] and atrophy of myotubes in vitro [37]. The negative effects of PA on myotubes can be attenuated by treatment with PUFA [37]. We also found that the levels of AA were significantly lower in patients with PM/DM compared to HI. The reduced AA levels are most likely due to the increased conversion of AA to pro-inflammatory prostaglandins and leukotrienes by cyclooxygenases and 5-lipoxygenase, in line with our previous observations of the enhanced expression of these enzymes in muscles from patients with PM and DM [5, 6].

We also found that the levels of nine lipid species within PC and TG were significantly altered in patients with PM/DM. Moreover, the levels of three PC and two TG species containing PUFA were lower and the levels of four TG species containing SFA and MUFA were higher in patients with PM/DM compared to HI. These data are in line with previous studies reporting that patients with rheumatic disease have lower levels of PUFAs and higher levels of SFA in serum PC, erythrocyte PL, and adipose tissue compared with HI [38–40]. These disproportionate PUFA and SFA levels that are increased with disease duration or activity are assumed to be related to the degree of inflammation and not to dietary habits [38, 40]. Furthermore, since SFA and PUFA have been shown to promote and counter muscle wasting, respectively [11, 37], the observed enhanced levels of SFA and reduced levels of PUFA in patients with PM/DM might have negative effects on muscle performance.

Using a multivariate approach we identified five lipid species PC(38:4), PC(36:4), TG(48:5), TG(50:6), and PC(40:8) that are important for the separation of patients with PM/DM and HI. Even though our study is small, and the detected changes are moderate, the results suggest altered lipid metabolism in patients with PM/DM. Further studies are needed to clarify the role of those lipids in disease progression.

We also detected altered serum lipid profiles in patients newly diagnosed with PM/DM in response to conventional immunosuppressive treatment, including high doses of GC in combination with immunosuppressive drugs. GC are well-known to affect lipid metabolism [41], and the role of GCs in the dysregulation of lipid metabolism in skeletal muscle has recently been addressed [10, 42]. In mouse myotubes in vitro and in skeletal muscle ex vivo, GC treatment affects the expression of genes involved in lipid storage, mobilization and utilization [42]. In patients with PM and DM, immunosuppressive treatment including GC affects the expression of genes involved in lipid and FA metabolism in skeletal muscle [10] which potentially might lead to altered lipid and FA profiles in those patients. Indeed, after conventional treatment we observed significantly higher levels of EDA and EPA. Minor catabolite of linoleic (18:2, n-6) acid, EDA and its subsequent metabolite sciadonic (20:3, n-6) acid may exert anti-inflammatory effects, affecting prostaglandin E_2_ (PGE_2_) and nitric oxide (NO) pathways [43]. EPA gives rise to lipid mediators that are less inflammatory than those produced from AA or involved in resolution of inflammation [43–45]. Consequently, the increased serum EPA and EDA levels after treatment might have a beneficial anti-inflammatory effect.

Moreover, the levels of 22 lipid species within PC, PE, LPC and TG were significantly altered after immunosuppressive treatment. However, we did not find a clear pattern in the changes of PUFA-enriched or SFA-enriched lipid species in response to the treatment indicating beneficial or detrimental treatment effects. Thirteen species were identified by MVA to be important for the separation of patients with PM/DM before and after treatment, i.e. PC(32:3), PC(32:2), PC(34:5), PC(34:4), PC(34:3), PC(36:6), PC(36:5), PE(32:2), PE(32:1), PE(34:3), PE(36:5), LPC(18:3) and LPC(20:5). We have observed correlation between s-CK and four lipid species, PE(36:1), PE(38:7), TG(56:7) and LPC(20:4), before and after treatment, suggesting lipid involvement in inflammatory processes. Furthermore, the correlation between FI and several lipid species detected before and after treatment indicates changes in lipid profiles of relevance for muscle performance.

These findings provide new evidence of the dysregulation of lipid metabolism in patients with PM/DM that may contribute to the persistent muscle weakness and fatigue. Interestingly, there is increasing evidence that dietary FA supplementation and physical exercise could be promising approaches to improve lipid profiles and muscle function [17, 18, 46, 47].

Though positive correlation between the proportions of individual PUFA in serum phospholipids and skeletal muscle phospholipids has been demonstrated in HI previously [48], future studies are needed to define to what extent the serum lipid profiles reflect the lipid profiles in skeletal muscle of patients with PM/DM.

Our current study has several limitations: one is the small number of patients included and the heterogeneity in the immunosuppressive treatment used in combination with GC, although all patients were treated with high doses of GC. In addition, it is not possible to differentiate between the relative effects of the disease progression and the immunosuppressive treatment on the lipid profiles. Another limitation is that we used the Bohan and Peter classification for PM and DM in this study due to lack of detailed information on the pattern of muscle weakness required to apply the 2017 European League Against Rheumatism (EULAR)/American College of Rheumatology (ACR) classification criteria for myositis [49]. Autoantibodies were positive in most patients as a support for an idiopathic inflammatory myopathy as compared to other myopathies. Moreover, patients with inclusion body myositis (IBM) or with histopathological features compatible with necrotizing myopathies were excluded from this study. Notably two individuals had anti-SRP or anti-HMGCR antibodies, both antibodies associated with IMNM, but none of these patients had clinical or histopathological features compatible with IMNM, one was classified as having PM and one as having DM, based on a combination of clinical and laboratory features [50]. We also did not have access to data on factors such as patient diet, lifestyle activity and disease activity. The serum samples from the patients in cohort 2 were collected before the suggested measures of disease activity from the International Myositis Assessment and Clinical Studies Group (IMACS) were available. Therefore we can only present a proxy of disease activity through measure of muscle performance Functional Index (FI) and results of s-CK levels for these patients. It would be of great interest to establish correlation between lipid changes and clinical outcome. Future studies in large cohorts are needed to clarify differences in lipid profiles and outcome measures in responders and non-responders to certain interventions.

## Conclusion

We have observed that serum lipids profiles are significantly altered in patients with PM/DM compared to age and gender matched HI. In addition, we observed that serum lipid profiles in patients newly diagnosed with PM/DM were significantly altered in response to immunosuppressive treatment. These findings suggest that lipid metabolism is dysregulated in patients with PM/DM and is affected by immunosuppressive treatment. Future studies will clarify whether the changes in serum lipid profiles reflect the muscle tissue lipid profiles and could be used as prognostic or therapeutic markers in PM/DM.

## Additional file

Additional file 1: **Table S1.** Lipid profiles in serum from patients with PM/DM and healthy individuals. **Table S2.** Lipid profiles in serum from patients with PM or DM before and after immunosuppressive treatment. (DOCX 44 kb)

## Abbreviations

AA: Arachidonic acid
anti-HMGCR: anti-3-Hydroxy-3-methylglutaryl-coenzyme A reductase
BF_3_: Boron trifluoride
CHCl_3_: Chloroform
DM: Dermatomyositis
EDA: Eicosadienoic acid
EPA: Eicosapentaenoic acid
ER: Endoplasmic reticulum
FA: Fatty acids
FAME: Fatty acid methyl ester
FI: Functional Index
GC: Glucocorticoids
GC-FID: Gas-chromatography flame ionization detector
HI: Healthy individuals
IMACS: International Myositis Assessment and Clinical Studies Group
IMNM: Immune necrotizing myopathy
LC-MS/MS: Liquid chromatography tandem mass spectrometry
LPC: Lysophosphatidylcholine
MeOH: Methanol
MRM: Multiple reaction monitoring
MUFA: Monounsaturated fatty acids
MVA: Multivariate analysis
OPLS: Orthogonal projection to latent structures
PA: Palmitic acid
PC: Phosphatidylcholine
PCA: Principal component analysis
PE: Phosphatidylethanolamine
PM: Polymyositis
PUFA: Polyunsaturated fatty acids
RA: Rheumatoid arthritis
s-CK: Creatine kinase
SD: Standard deviation
SFA: Saturated fatty acids
SPH: Sphingomyelin
TG: Triacylglycerol
TSH: Thyroid stimulating hormone
VIP: Variable influence in projection
