# Supplementary material for: Targeted lipidomics analysis identified altered serum lipid profiles in patients with polymyositis and dermatomyositis
Source: Arthritis Res Ther. 2018 May 2;20:83. doi: 10.1186/s13075-018-1579-y (PMC5932839; doi:10.1186/s13075-018-1579-y)
Supplement: Supplementary file 1 — Table S1. Lipid profiles in serum from patients with PM/DM and healthy individuals. Table S2. Lipid profiles in serum from patients with PM or DM before and after immunosuppressive treatment. (DOCX 44 kb) [file 13075_2018_1579_MOESM1_ESM.docx]

**Table S1. Lipid profiles in serum from patients with PM/DM and healthy individuals.**

| **Lipid species** | **Healthy individuals**  **area %** | **PM/DM patients**  **area%** | **p-value** |
| --- | --- | --- | --- |
| PC(32:3) | 0.02 ± 0.01 | 0.02 ± 0.01 | 0.644 |
| PC(32:2) | 0.30 ± 0.08 | 0.38 ± 0.15 | 0.126 |
| PC(32:1) | 1.71 ± 0.82 | 2.21 ± 1.19 | 0.207 |
| PC(32:0) | 0.02 ± 0.01 | 0.02 ± 0.01 | 0.644 |
| PC(34:5) | 0.01 ± 0.01 | 0.02 ± 0.01 | 0.264 |
| PC(34:4) | 0.10 ± 0.05 | 0.13 ± 0.06 | 0.254 |
| PC(34:3) | 1.54 ± 0.33 | 1.87 ± 0.75 | 0.120 |
| PC(34:2) | 26.34 ± 2.04 | 27.66 ± 2.95 | 0.190 |
| PC(34:1) | 16.29 ± 1.90 | 17.38 ± 2.39 | 0.244 |
| PC(34:0) | 1.44 ± 0.16 | 1.55 ± 0.22 | 0.099 |
| PC(36:6) | 0.07 ± 0.03 | 0.08 ± 0.04 | 0.452 |
| PC(36:5) | 3.19 ± 1.19 | 3.16 ± 2.25 | 0.356 |
| PC(36:4) | 9.34 ± 1.15 | 8.17 ± 1.19* | 0.0116 |
| PC(36:3) | 8.29 ± 0.94 | 7.60 ± 1.16 | 0.089 |
| PC(36:2) | 12.15 ± 1.37 | 12.12 ± 1.13 | 0.662 |
| PC(36:1) | 2.62 ± 0.35 | 2.57 ± 0.45 | 0.560 |
| PC(36:0) | 0.28 ± 0.04 | 0.28 ± 0.05 | 0.593 |
| PC(38:9) | 0.33 ± 0.08 | 0.31 ± 0.09 | 0.395 |
| PC(38:8) | 0.41 ± 0.06 | 0.36 ± 0.09 | 0.0584 |
| PC(38:7) | 0.16 ± 0.04 | 0.14 ± 0.07 | 0.0726 |
| PC(38:6) | 5.35 ± 1.19 | 5.26 ± 1.26 | 0.999 |
| PC(38:5) | 3.77 ± 0.46 | 3.28 ± 0.91* | 0.0133 |
| PC(38:4) | 4.11 ± 0.66 | 3.41 ± 0.49* | 0.0198 |
| PC(40:10) | 0.02 ± 0.01 | 0.02 ± 0.01 | 0.409 |
| PC(40:9) | 0.11 ± 0.02 | 0.10 ± 0.03 | 0.560 |
| PC(40:8) | 0.06 ± 0.01 | 0.05 ± 0.02 | 0.0688 |
| PC(40:7) | 0.35 ± 0.07 | 0.31 ±0.08 | 0.145 |
| PC(40:6) | 1.20 ± 0.29 | 1.17 ± 0.34 | 0.922 |
| PC(42:11) | 0.04 ± 0.01 | 0.04 ± 0.01 | 0.884 |
| PC(44:12) | 0.35 ± 0.07 | 0.31 ± 0.07 | 0.145 |
| PC_S/P | 2.1+0.3 | 2.5+0.6* | 0.0369* |
| LPC(14:0) | 0.54 ± 0.23 | 0.55 ± 0.21 | 0.715 |
| LPC(16:1) | 1.18 ± 0.30 | 1.23 ± 0.31 | 0.627 |
| LPC(16:0) | 37.99 ± 3.30 | 36.21 ± 4.72 | 0.120 |
| LPC(18:4) | 0.002 ± 0.002 | 0.01 ± 0.01 | 0.114 |
| LPC(18:3) | 0.50 ± 0.13 | 0.58 ± 0.17 | 0.234 |
| LPC(18:2) | 23.46 ± 3.58 | 24.45 ± 3.41 | 0.356 |
| LPC(18:1) | 14.34 ± 1.20 | 15.52 ± 1.86 | 0.099 |
| LPC(18:0) | 12.00 ± 2.16 | 11.58 ± 1.57 | 0.593 |
| LPC(20:5) | 1.47 ± 0.71 | 1.55 ± 1.38 | 0.409 |
| LPC(22:6) | 2.50 ± 0.77 | 2.69 ± 0.94 | 0.593 |
| LPC(20:4) | 6.03 ± 1.13 | 5.62 ± 1.13 | 0.356 |
| TG(42:0) | 0.27 ± 0.32 | 0.20 ± 0.20 | 0.77 |
| TG(44:1) | 0.36 ± 0.37 | 0.24 ± 0.37 | 0.234 |
| TG(44:0) | 0.24 ± 0.28 | 0.63 ± 0.78 | 0.382 |
| TG(46:1) | 1.58 ± 1.69 | 2.38 ± 1.24* | 0.0174 |
| TG(46:0) | 0.99 ± 0.85 | 1.78 ± 1.10 t | 0.0765 |
| TG(48:5) | 0.30 ± 0.15 | 0.14 ± 0.10** | 0.0036 |
| TG(48:4) | 0.53 ± 0.33 | 0.48 ± 0.22 | 0.865 |
| TG(48:2) | 1.67 ± 0.95 | 2.35 ± 0.95* | 0.0369 |
| TG(48:1) | 2.03 ± 1.40 | 3.16 ± 1.10* | 0.0101 |
| TG(48:0) | 1.77 ± 0.83 | 2.40 ± 0.96 | 0.0584 |
| TG(50:6) | 0.26 ± 0.20 | 0.15 ± 0.10 | 0.166 |
| TG(50:5) | 0.41 ± 0.28 | 0.41 ± 0.19 | 0.770 |
| TG(50:2) | 5.23 ± 1.15 | 6.07 ± 1.51 | 0.126 |
| TG(50:1) | 7.64 ± 1.86 | 9.53 ± 2.39* | 0.0290 |
| TG(52:6) | 0.45 ± 0.25 | 0.45 ± 0.25 | 0.865 |
| TG(52:5) | 1.06 ± 0.54 | 0.91 ± 0.30 | 0.274 |
| TG(52:4) | 4.30 ± 0.64 | 4.24 ± 0.63 | 0.770 |
| TG(52:3) | 14.52 ± 1.74 | 13.73 ± 1.72 | 0.198 |
| TG(52:2) | 28.66 ± 3.01 | 27.05 ± 3.55 | 0.159 |
| TG(54:7) | 0.49 ± 0.26 | 0.39 ± 0.22 | 0.344 |
| TG(54:6) | 1.19 ± 0.31 | 1.26 ± 0.52 | 0.770 |
| TG(54:5) | 5.19 ± 0.84 | 4.35 ± 1.44 | 0.0894 |
| TG(54:4) | 8.88 ± 1.90 | 7.27 ± 1.84* | 0.0256 |
| TG(54:3) | 10.31 ± 2.54 | 9.01 ± 2.00 | 0.159 |
| TG(56:7) | 1.68 ± 0.59 | 1.40 ± 0.99 | 0.0990 |
| TG_S/P | 1.0+0.3 | 1.3+0.3* | 0.0327 |
| SPH(16:0) | 42.19 ± 14.11 | 37.74 + 13.50 | 0.285 |
| SPH(18:3) | 0.002±0.004 | 0.002±0.004 | 0.789 |
| SPH(18:2) | 0.32 ± 0.08 | 0.35 + 0.16 | 0.846 |
| SPH(18:1) | 12.70 ± 3.37 | 13.66 + 3.36 | 0.437 |
| SPH(18:0) | 43.35 ± 10.30 | 48.21 + 10.47 | 0.190 |
| SPH(20:4) | 0.06 ± 0.03 | 0.04 + 0.04 | 0.0894 |

HI, healthy individuals; PM, polymyositis; DM, dermatomyositis; LPC, lysophosphatidylcholine; PC, phosphatidylcholine; PS, polyunsaturated species; SPH, sphingomyelin; S, saturated species; S/P, ratio of the species containing saturated and monounsaturated fatty acids to the species containing polyunsaturated fatty acids; TG, triacylglycerol; Values given as mean ± SD. *p<0.05. **p<0.01, PM/DM patients *vs* HI.

**Table S2. Lipid profiles in serum from patients with PM or DM before and after immunosuppressive treatment.**

| **Lipid species** | **Before treatment area%** | **After treatment area%** | **p-value** |
| --- | --- | --- | --- |
| PC(32:3) | 0.03 ± 0.01 | 0.05 ± 0.02* | 0,0357 |
| PC(32:2) | 0.77 ± 0.18 | 0.98 ± 0.17* | 0,0117 |
| PC(32:1) | 3.02 ± 1.17 | 4.38 ± 1.40* | 0,0173 |
| PC(32:0) | 1.18 ± 0.15 | 1.06 ± 0.17* | 0,0357 |
| PC(34:5) | 0.01 ± 0.01 | 0.04 ± 0.02* | 0,0117 |
| PC(34:4) | 0.12 ± 0.05 | 0.30 ± 0.11* | 0,0117 |
| PC(34:3) | 2.29 ± 0.41 | 3.76 ± 0.76* | 0,0117 |
| PC(34:2) | 25.42 ± 3.41 | 24.08 ± 2.78 | 0,207 |
| PC(34:1) | 17.67 ± 2.00 | 17.72 ± 3.03 | 0,888 |
| PC(34:0) | 1.58 ± 0.23 | 1.67 ± 0.28 | 0,327 |
| PC(36:6) | 0.10 ± 0.05 | 0.22 ± 0.08* | 0,0117 |
| PC(36:5) | 3.18 ± 1.00 | 4.64 ± 1.51* | 0,0251 |
| PC(36:4) | 7.09 ± 1.47 | 6.32 ± 1.15 | 0,0929 |
| PC(36:3) | 5.25 ± 1.32 | 4.79 ± 1.10 | 0,400 |
| PC(36:2) | 12.39 ± 1.31 | 11.84 ± 1.21 | 0,327 |
| PC(36:1) | 3.42 ± 0.62 | 3.93 ± 0.85 | 0,123 |
| PC(36:0) | 0.38 ± 0.09 | 0.43 ± 0.09 | 0,207 |
| PC(38:9) | 0.23 ± 0.03 | 0.21 ± 0.03 | 0,123 |
| PC(38:8) | 0.49 ± 0.06 | 0.52 ± 0.13 | 0,575 |
| PC(38:7) | 0.16 ± 0.04 | 0.19 ± 0.07 | 0,483 |
| PC(38:6) | 6.61 ± 1.64 | 5.42 ± 1.44 | 0,068 |
| PC(38:5) | 2.55 ± 0.52 | 2.70 ± 0.81 | 0,483 |
| PC(38:4) | 3.65 ± 0.99 | 2.91 ± 0.98* | 0,0357 |
| PC(40:10) | 0.02 ± 0.01 | 0.02 ± 0.01 | 0,128 |
| PC(40:9) | 0.16 ± 0.05 | 0.11 ± 0.05 | 0,123 |
| PC(40:8) | 0.03 ± 0.01 | 0.04 ± 0.01 | 0,107 |
| PC(40:7) | 0.38 ± 0.09 | 0.31 ± 0.08* | 0,0117 |
| PC(40:6) | 1.80 ± 0.58 | 1.36 ± 0.40* | 0,0251 |
| PC(42:11) | 0.00 ± 0.001 | 0.003 ± 0.001 | 0,0519 |
| PC(44:12) | 0.00 ± 0.0005 | 0.001 ± 0.0004 | 0,463 |
| PE(32:2) | 0.02 ± 0.01 | 0.04 ± 0.02 | 0,123 |
| PE(32:1) | 0.30 ± 0.18 | 0.58 ± 0.29* | 0,0173 |
| PE(32:0) | 0.11 ± 0.04 | 0.15 ± 0.04 | 0,207 |
| PE(34:5) | 0.11 ± 0.04 | 0.14 ± 0.04 | 0,207 |
| PE(34:4) | 0.02 ± 0.02 | 0.02 ± 0.004 | 0,327 |
| PE(34:3) | 0.42 ± 0.16 | 0.83 ± 0.29* | 0,0117 |
| PE(34:2) | 9.33 ± 1.68 | 11.23 ± 2.53 | 0,0687 |
| PE(34:1) | 5.38 ± 1.29 | 5.39 ± 1.07 | 0,999 |
| PE(34:0) | 0.78 ± 0.88 | 0.47 ± 0.09 | 0,207 |
| PE(36:6) | 0.05 ± 0.03 | 0.07 ± 0.02* | 0,0423 |
| PE(36:5) | 0.76 ± 0.32 | 1.42 ± 0.40* | 0,0117 |
| PE(36:4) | 9.15 ± 2.11 | 8.61 ± 2.42 | 0,123 |
| PE(36:3) | 6.41 ± 2.66 | 7.03 ± 0.77 | 0,575 |
| PE(36:2) | 19.64 ± 5.29 | 21.00 ± 2.49 | 0,483 |
| PE(36:1) | 2.17 ± 0.46 | 2.20 ± 0.33 | 0,674 |
| PE(36:0) | 1.92 ± 2.05 | 1.56 ± 0.57 | 0,483 |
| PE(38:9) | 0.04 ± 0.02 | 0.04 ± 0.01 | 0,528 |
| PE(38:8) | 0.03 ± 0.01 | 0.03 ± 0.02 | 0,888 |
| PE(38:7) | 0.13 ± 0.07 | 0.14 ± 0.06 | 0,340 |
| PE(38:6) | 15.36 ± 4.38 | 14.88 ± 3.54 | 0,575 |
| PE(38:5) | 5.65 ± 0.87 | 5.91 ± 1.49 | 0,400 |
| PE(38:4) | 15.66 ± 3.33 | 13.09 ± 2.76* | 0,0357 |
| PE(40:10) | 0.01 ± 0.004 | 0.01 ± 0.01 | 0,176 |
| PE(40:9) | 0.03 ± 0.01 | 0.05 ± 0.03 | 0,0687 |
| PE(40:8) | 0.08 ± 0.03 | 0.08 ± 0.02 | 0,575 |
| PE(40:7) | 1.68 ± 0.45 | 1.42 ± 0.34 | 0,0117 |
| PE(40:6) | 4.76 ± 1.79 | 3.58 ± 1.15* | 0,0173 |
| PE(42:11) | 0.01 ± 0.01 | 0.01 ± 0.01 | 0,234 |
| PE(44:12) | 0.01 ± 0.01 | 0.01 ± 0.01 | 0,916 |
| LPC(14:0) | 0.94 ± 1.08 | 0.93 ± 0.28 | 0,161 |
| LPC(16:1) | 1.26 ± 0.40 | 1.68 ± 0.60* | 0,0173 |
| LPC(16:0) | 49.00 ± 6.80 | 46.60 ± 6.05 | 0,207 |
| LPC(18:3) | 0.32 ± 0.21 | 0.68 ± 0.36* | 0,0499 |
| LPC(18:2) | 15.64 ± 5.51 | 17.42 ± 4.98 | 0,327 |
| LPC(18:1) | 12.76 ± 1.93 | 14.20 ± 3.34 | 0,207 |
| LPC(18:0) | 15.10 ± 2.49 | 13.71 ± 3.02 | 0,262 |
| LPC(20:5) | 0.52 ± 0.21 | 0.78 ± 0.20* | 0,0173 |
| LPC(22:6) | 1.59 ± 0.60 | 1.38 ± 0.24 | 0,400 |
| LPC(20:4) | 2.86 ± 1.09 | 2.62 ± 0.86 | 0,575 |
| TG(44:1) | 2.17 ± 2.16 | 3.21 ± 2.44 | 0,483 |
| TG(44:0) | 1.94 ± 1.81 | 1.98 ± 1.87 | 0,99 |
| TG(46:1) | 4.50 ± 3.24 | 5.45 ± 2.50 | 0,575 |
| TG(46:0) | 2.89 ± 1.80 | 3.59 ± 1.42 | 0,483 |
| TG(48:5) | 0.34 ± 0.57 | 0.17 ± 0.11 | 0,779 |
| TG(48:4) | 1.26 ± 1.00 | 1.21 ± 0.82 | 0,674 |
| TG(48:2) | 2.92 ± 1.07 | 3.90 ± 0.92 | 0,123 |
| TG(48:1) | 3.23 ± 1.49 | 4.57 ± 1.09* | 0,049 |
| TG(48:0) | 2.75 ± 2.08 | 2.80 ± 1.44 | 0,999 |
| TG(50:6) | 0.62 ± 1.13 | 0.33 ± 0.18 | 0,779 |
| TG(50:5) | 0.65 ± 0.60 | 0.82 ± 0.36 | 0,483 |
| TG(50:2) | 4.71 ± 0.84 | 5.32 ± 1.59 | 0,483 |
| TG(50:1) | 7.26 ± 1.69 | 7.72 ± 1.43 | 0,674 |
| TG(52:6) | 0.33 ± 0.30 | 0.49 ± 0.23 | 0,400 |
| TG(52:5) | 0.79 ± 0.40 | 0.75 ± 0.28 | 0,575 |
| TG(52:4) | 3.68 ± 1.19 | 3.45 ± 0.40 | 0,483 |
| TG(52:3) | 11.39 ± 3.27 | 10.03 ± 1.68 | 0,327 |
| TG(52:2) | 20.31 ± 4.11 | 20.00 ± 4.53 | 0,575 |
| TG(54:7) | 0.42 ± 0.35 | 0.30 ± 0.23 | 0,483 |
| TG(54:6) | 1.25 ± 0.44 | 1.12 ± 0.39 | 0,779 |
| TG(54:5) | 5.76 ± 1.83 | 4.87 ± 1.85 | 0,262 |
| TG(54:4) | 9.71 ± 3.86 | 8.82 ± 3.50 | 0,327 |
| TG(54:3) | 9.13 ± 2.41 | 7.42 ± 3.13 | 0,161 |
| TG(56:7) | 1.98 ± 1.19 | 1.69 ± 0.86 | 0575 |

PM, polymyositis; DM, dermatomyositis; LPC, lysophosphatidylcholine; PC, phosphatidylcholine; PE, phosphatidylethanolamine; PS, polyunsaturated species; S, saturated species; S/P, ratio of the species containing saturated and monounsaturated fatty acids to the species containing polyunsaturated fatty acids; TG, triacylglycerol; Values given as mean ± SD. *p<0.05, PM/DM patients after *vs* before treatment.
